# Production of glycoprotein vaccines in *Escherichia coli*

**DOI:** 10.1186/1475-2859-9-61

**Published:** 2010-08-11

**Authors:** Julian Ihssen, Michael Kowarik, Sandro Dilettoso, Cyril Tanner, Michael Wacker, Linda Thöny-Meyer

**Affiliations:** 1 Empa, Swiss Federal Laboratories for Materials Testing and Research, Laboratory for Biomaterials, Lerchenfeldstrasse 5, CH-9014 St. Gallen, Switzerland; 2 GlycoVaxyn AG, Grabenstrasse 3, CH-8952 Schlieren, Switzerland

## Abstract

**Background:**

Conjugate vaccines in which polysaccharide antigens are covalently linked to carrier proteins belong to the most effective and safest vaccines against bacterial pathogens. State-of-the art production of conjugate vaccines using chemical methods is a laborious, multi-step process. *In vivo *enzymatic coupling using the general glycosylation pathway of *Campylobacter jejuni *in recombinant *Escherichia coli *has been suggested as a simpler method for producing conjugate vaccines. In this study we describe the *in vivo *biosynthesis of two novel conjugate vaccine candidates against *Shigella dysenteriae *type 1, an important bacterial pathogen causing severe gastro-intestinal disease states mainly in developing countries.

**Results:**

Two different periplasmic carrier proteins, AcrA from *C. jejuni *and a toxoid form of *Pseudomonas aeruginosa *exotoxin were glycosylated with *Shigella *O antigens in *E. coli*. Starting from shake flask cultivation in standard complex medium a lab-scale fed-batch process was developed for glycoconjugate production. It was found that efficiency of glycosylation but not carrier protein expression was highly susceptible to the physiological state at induction. After induction glycoconjugates generally appeared later than unglycosylated carrier protein, suggesting that glycosylation was the rate-limiting step for synthesis of conjugate vaccines in *E. coli*. Glycoconjugate synthesis, in particular expression of oligosaccharyltransferase PglB, strongly inhibited growth of *E. coli *cells after induction, making it necessary to separate biomass growth and recombinant protein expression phases. With a simple pulse and linear feed strategy and the use of semi-defined glycerol medium, volumetric glycoconjugate yield was increased 30 to 50-fold.

**Conclusions:**

The presented data demonstrate that glycosylated proteins can be produced in recombinant *E. coli *at a larger scale. The described methodologies constitute an important step towards cost-effective *in vivo *production of conjugate vaccines, which in future may be used for combating severe infectious diseases, particularly in developing countries.

## Background

**Figure 1:**
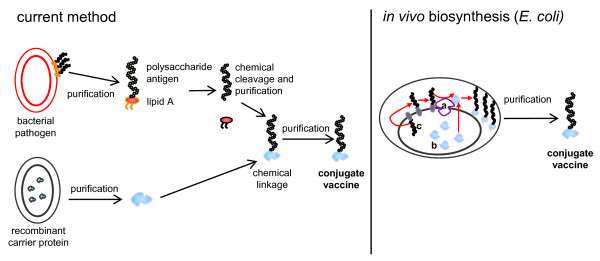
**Current method for the production of conjugate vaccines and *in vivo *biosynthesis**. a: oligosaccharyltransferase PglB, b: carrier protein with signal sequence for secretion to the periplasm, c: undecaprenyl-pyrophosphate-linked polysaccharides.

In conjugate vaccines capsular or lipopolysaccharide (LPS) antigens of pathogenic bacteria are covalently bound to carrier proteins [1-3]. In contrast to isolated bacterial polysaccharides, conjugate vaccines induce a long-lasting T-lymphocyte dependent immunological memory [4,5]. Efficacy and safety of conjugate vaccines have been proven for several examples (reviewed by [3] and [5]). Most notably routine immunization of infants with conjugate vaccines against *Haemophilus influenzae *type B led to a fast and dramatic drop in respective disease incidents after implementation. State-of-the art production technologies for conjugate vaccines are complex, multi-step processes (Figure 1). They involve (i) separate cultivation of bacterial strains producing the polysaccharide antigens and the carrier protein, (ii) separate purification of LPS and carrier protein, (iii) chemical cleavage of LPS polysaccharides from lipid A followed by a second purification step, (iv) chemical coupling of polysaccharides to the carrier protein, and (v) a third purification step for obtaining the final product [1,2]. At each step considerable losses occur, and due to the random nature of chemical coupling the final products are ill-defined. The processes are time-consuming and costly, and often large-scale cultivation of pathogenic bacteria is required for polysaccharide biosynthesis, making conjugate vaccines prohibitively expensive for vaccination campaigns in developing countries.

In recent years the notion that bacteria do not perform protein glycosylation has become obsolete [6-8]. After functional transfer of the general N-linked glycosylation system of *Campylobacter jejuni *into *Escherichia coli *it is now possible to produce polysaccharide-protein conjugates in a standard industrial prokaryotic expression host [8,9]. It has been shown that diverse bacterial O antigen polysaccharides with an N-acetyl sugar at the reducing end can be transferred from undecaprenyl-pyrophosphate precursors to the periplasmic protein AcrA (originating from *C. jejuni*) in *E. coli *[9,10]. Furthermore, the consensus sequence required for N-linked glycosylation by the oligosaccharyltransferase PglB of *C. jejuni *has been defined as D/E-X-N-Z-S/T (where X and Z can be any amino acid except proline) [11], making it possible to engineer specific glycosylation sites into proteins which are otherwise not glycosylated [12]. The PglB-based *E. coli *system has been suggested as a simple and cost-efficient method for *in vivo *production of conjugate vaccines. These conjugates were termed 'bioconjugates' to highlight the *in vivo *production process [9].

For achieving sufficient time-space yields of recombinant proteins produced with bacterial expression systems it is usually necessary to reach high final cell densities in bioprocesses. Although plasmid-free, wild-type *E. coli *can be grown to very high biomass concentrations of 100 g to 170 g cell dry weight per liter in fed-batch culture with defined mineral salts media [13,14], to reach high titers of correctly folded recombinant proteins with plasmid-bearing strains remains a challenge.

In this work, we describe the establishment of an efficient and reproducible fed-batch process for the *in vivo *production of two novel glycoconjugates composed of the *Shigella dysenteriae *serotype 1 O antigen and carrier proteins AcrA *of C. jejuni *and exotoxin A of *P. aeruginosa *(EPA). The bioconjugates are potential vaccines against shigellosis.

Shigellosis is estimated to cause 163 illness episodes and 1 million deaths per year in poor countries, with children under the age of five being particularly affected [15,16]. There is an urgent need for efficient multivalent vaccines to combat this disease [17]. Shigellosis is caused by four major *Shigella *species, *S. dysenteriae*, *S. flexneri*, *S. boydii *and *S. sonnei*. Although *S. dysenteriae *serotype 1 is not among the most common clinical isolates, it is desirable to include the respective polysaccharide antigen in multivalent vaccine formulations because the strain is associated with a high rate of case fatality, pandemic spread and multiple antibiotic resistance [15].

## Methods

### Bacterial Strains and plasmids

*Escherichia coli *CLM24 [9] was used as host strain in all experiments. This strain was derived from W3110 by deletion of the chromosomal gene coding for the O polysaccharide ligase WaaL [9]. Ampicillin-selectable, medium copy number plasmid pMIK44 [11] was used for periplasmic expression of AcrA under the control of the L-arabinose (ara) inducible promoter P_*BAD *_(origin of replication: ColE1). Recombinant AcrA expressed from pMIK44 contains 2 native and 1 engineered N-glycosylation sites and is fused to a hexahistidine tag at the C-terminal end and to the PelB signal peptide at the N-terminal end (Sec-dependent secretion to the periplasm). Ampicillin-selectable, medium copy number plasmid pGVXN150 (provided by GlycoVaxyn AG, manuscript in preparation) was used for periplasmic expression of a toxoid variant (L552V, ΔE553) of *P. aeruginosa *exotoxin A (EPA) under the control of P_*BAD *_(origin of replication: ColE1). Recombinant EPA expressed from pGVXN150 contains two engineered N-glycosylation sites (N262 and N398) and is fused to a hexahistidine tag at the C-terminal end and to the DsbA signal peptide at the N-terminal end (Sec-dependent secretion to the periplasm). Tetracycline-selectable, low copy number plasmid pGVXN64 was used for biosynthesis of O antigen polysaccharides of *S. dysenteriae *serotype 1. pGVXN64 (origin of replication: IncPα) was constructed by insertion of an 11 kb *Bam*HI fragment of pSDM7 [18] containing the *S. dysenteriae rfp *and *rfb *gene clusters into the *Bam*HI site of pLAFR1 [19,20]. The *rfp *and *rfb *gene clusters encode glycosyltransferases and polymerases required for the synthesis of undecaprenyl-pyrophosphate-linked *Shigella *O1 polysaccharides [18] and were expressed from their native (constitutive) promoters in pGVXN64.

Spectinomycin-selectable, low copy number plasmid pGVXN114 was used for expression of oligosaccharyltransferase PglB from *C. jejuni *under the control of the hybrid P_*tac *_promoter which can be induced by isopropyl-β-D-thiogalactopyranoside (origin of replication: IncW). Recombinant PglB expressed from pGVXN114 contains a hemagglutinin (HA) oligopeptide tag at the C-terminal end in order to facilitate its detection on Western blots. The LacI repressor was constitutively expressed from the same plasmid. pGVXN114 was constructed by insertion of a 2.2 kb *Eco*RI-*Bam*HI fragment of pMAF10 [9] into pEXT21 [21] digested with *Eco*RI and *Bam*HI. Spectinomycin-resistant plasmid pGVXN115 was used for IPTG inducible expression of inactive PglBmut (amino acid substitutions W458A and D459A). pGVXN115 was constructed by insertion of a 2.2 kb *Eco*RI-*Bam*HI fragment of pWA1 [9] into pEXT21 digested with *Eco*RI and *Bam*HI.

### Shake flask experiments

For biosynthesis of glycoconjugates in shake flasks, recombinant *E. coli *containing plasmids for expression of carrier protein, *Shigella *O1 polysaccharides and PglB were grown in LB medium (10 g L^-1 ^casein-based tryptone, 5 g L^-1 ^yeast extract, 5 g L^-1 ^NaCl) supplemented with 100 mg L^-1^ampicillin, 10 mg L^-1 ^tetracycline and 80 mg L^-1 ^spectinomycin at 37°C and at an agitation of 160 rpm. Shake flask cultures were prepared with a low surface to volume ratio (70 mL medium in 100 mL Erlenmeyer flasks) and were inoculated from an uninduced LB overnight culture to an OD_600 _of 0.05 to 0.1. Expression of PglB and carrier protein (ArcA, EPA) was induced at an OD_600 _of 0.4 to 0.5 by the addition of 1 mM isopropyl-β-D-thiogalactopyranoside (IPTG) and 2 g L^-1 ^L-arabinose, respectively. IPTG was added as 1000 × concentrated solution (1 mL L^-1 ^of 1 M) and L-arabinose as 200 × concentrated solution (5 mL L^-1 ^of 400 g L^-1^). Four hours after the first induction, a second pulse of 2 g L^-1 ^L-arabinose was added. Samples for Western blot analysis were withdrawn 4 h after the first induction and after overnight incubation (total incubation time 20-24 h, total induction time 19-22 h). The effect of reduced inducer concentrations was analyzed in parallel shake flask cultures where the added concentration of IPTG was 1000 μM, 50 μM, 20 μM and 5 μM, respectively. The added amounts of L-arabinose were not changed. For testing the effect of reduced cultivation temperature, shake flask cultures were grown at 30°C to an OD_600 _of 0.4 to 0.5 and then induced with either 1 mM or 50 μM IPTG. After induction, the incubation temperature was reduced further to 23°C. The added amounts of L-arabinose were the same as in experiments performed at 37°C.

Inoculum for bioreactors was produced by cultivating recombinant *E. coli *strains overnight at 37°C and 150 rpm in LB medium with antibiotics using 500 mL baffled flasks (200 mL liquid volume). A final OD_600 _of 2.5 - 3.0 was reached in these cultures.

For growth tests in medium without complex supplements a defined carbon-limited mineral salts medium was used with 4 g L^-1 ^glucose as sole source of carbon and energy [22]. Three replicate shake flasks (total volume 300 mL, liquid volume 50 mL) were inoculated to an OD_600 _of 0.025 with uninduced cells from overnight LB cultures which had been washed twice with pre-warmed mineral medium. Flask cultures were incubated at 37°C and 150 rpm and specific growth rates were calculated from OD_600 _values between 0.1 and 0.8 measured after a pre-incubation period of 3 h. In this OD range logarithmic growth curves were linear.

### Bioreactor experiments

For larger-scale cultivation of recombinant *E. coli*, either MCS11 bioreactors (MBR, Wetzikon, Switzerland) with a total volume of 2.5 L and 3.5 L or a KLF bioreactor (Bioengineering, Wald, Switzerland) with a total volume of 3.5 L were used. Temperature was always controlled at 37(± 0.1)°C.

Batch cultivations were performed with *in situ *autoclaved LB medium supplemented with 100 μg mL^-1 ^ampicillin, 10 μg mL^-1 ^tetracycline, 80 μg mL^-1^spectinomycin and 0.2 mL L^-1 ^polypropylene glycol (PPG, antifoam agent). The liquid volume was 1.5 L, the stirrer speed was set to 1000 rpm and the bioreactor was aerated with an air flow of 1.4 L L^-1 ^min^-1 ^(resulting in pO_2 _≥ 60%). Bioreactor batch cultures were inoculated with uninduced overnight LB shake flask cultures to an OD_600 _of 0.05. Expression of recombinant proteins was induced at an OD_600 _of 0.5 by adding 1 mM IPTG and 2 g L^-1 ^L-arabinose.

For chemostat cultivation with LB medium an acidified feed solution was used which was composed of 5 g L^-1 ^yeast extract, 10 g L^-1 ^tryptone, 5 g l^-1 ^NaCl, 2.7 g l^-1 ^KH_2_PO_4 _and 0.1 ml l^-1 ^concentrated H_2_SO_4_. For testing the effect of alternative carbon- and energy sources, an acidified, semi-defined feed medium was used with the following composition: 10 g L^-1 ^glycerol or glucose, 1 g L^-1 ^yeast extract, 2 g L^-1 ^tryptone, 7.5 g L^-1 ^KH_2_PO_4_, 2.9 g L^-1 ^NH_4_Cl, 1 g L^-1 ^MgSO_4_·7H_2_O, 1 g L^-1 ^citric acid, 0.1 ml L^-1 ^HCl 37%, 1 mL L^-1 ^PPG antifoam and 10 mL L^-1 ^of 100 × trace element solution. 100 × trace element solution was composed of (added in this order): 8 mL L^-1 ^HCl 37%, 10 g L^-1 ^CaCO_3 _, 20 g L^-1 ^FeCl_3 _6 H_2_O, 1.5 g L^-1 ^MnCl_2 _4 H_2_O, 0.15 g L^-1 ^CuSO_4 _5 H_2_O, 0.25 g L^-1 ^CoCl_2 _6 H_2_O, 0.20 g L^-1 ^ZnSO_4_·7H_2_O, 0.30 10^-3 ^g L^-1 ^H_3_BO_3_, 2.0 g L^-1 ^NaMoO_4 _2 H_2_O, and 84.4 g L^-1 ^Na_4_EDTA 2 H_2_O (equimolar to cations). The concentration of mineral salts was chosen such that the carbon- and energy source was the growth-limiting nutrient. Respective calculations were based on growth yields for each element and the maximal biomass concentration supported by 10 g L^-1 ^glucose as described by Egli [23]. Antibiotics were added at similar concentrations as in medium for batch cultivation. To avoid any heat-induced reactions of LB components with phosphate salts or metal ions, chemostat feed media were sterilized by filtration (Sartobran 300 unit with sequential 0.4 μm and 0.2 μm pore sizes, Sartorius Stedim Biotech S.A., Aubagne, France). Depending on the extent of foaming in the bioreactor, heat-sterilized PPG antifoam was added in concentrations of 0.1 to 1 ml L^-1 ^to the feed tank. In chemostat experiments pH was controlled at 7.0 ± 0.05 by automated addition of 4 M KOH. The liquid volume was kept constant at 1.5 L by automated weight measurement of the reactor and the dilution rate was set to 0.1 h^-1^. Cultures were kept oxic (pO_2 _≥ 20%) by using an aeration rate of 1 L_air _L^-1 ^min^-1 ^and a stirrer speed of 1300 rpm. Chemostat cultures were inoculated to an OD_600 _of 0.05 with uninduced overnight LB shake flask cultures. Cells were grown in batch mode for 2-3 h to an OD_600 _of 0.6; at this time point the pump for the feed medium was turned on. Chemostat cultures were induced after 20 h (≈ 2 volume changes) by switching to a feed medium containing 2 g L^-1 ^L-arabinose in addition to the other components. After 4 h of arabinose induction the feed medium was switched back to the initial composition, and 1 mM IPTG was added directly into the reactor (sequential, separate induction of carrier protein and oligosaccharyltransferase).

For fed-batch cultivation with linear feed (strategy A) the starting medium (V = 1.5 L) contained 30 g L^-1 ^glycerol, 10 g L^-1 ^yeast extract, 20 g L^-1 ^tryptone, 10 g L^-1 ^KH_2_PO_4_, 5 g L^-1 ^(NH_4_)_2_SO_4_, 0.5 g L^-1 ^MgSO_4_·7H_2_O and 10 mL L^-1 ^of 100 × trace element solution (see above). Antibiotics were added in similar concentrations as in media for batch and chemostat cultivation. Prior to inoculation pH was adjusted to pH7 with 4 M KOH. At an OD_600 _of 24, a linear feed of 50 mL L^-1 ^h^-1 ^was started with feed solution A1 containing 240 g L^-1 ^glycerol, 72 g L^-1 ^tryptone, 1.5 g L^-1 ^MgSO_4_·7H_2_O and 10 mL L^-1 ^100 × trace element solution. After 1 h, when OD_600 _had reached 35, the feed rate was increased to 65 mL L^-1 ^h^-1^. After another 2 h, when OD_600 _had reached 47, cells were induced by adding 2 g L^-1 ^L-arabinose and 1 mM IPTG directly to the reactor, at the same time the linear feed was switched to feed solution A2 containing 48 g L^-1 ^L-arabinose and 80 μM IPTG in addition to the components of feed solution A1. The feed rate was reduced to 30 mL L^-1 ^h^-1 ^five hours later. The total added volumes of feed solutions A1 and A2 were 180 mL L^-1 ^and 975 mL^-1 ^L^-1^, respectively.

For fed-batch cultivation with two nutrient and inducer pulses (strategy B) the starting medium (V = 1.5 L) contained 30 g L^-1 ^glycerol, 5 g L^-1 ^yeast extract, 10 g L^-1 ^tryptone, 10 g L^-1 ^KH_2_PO_4_, 5 g L^-1 ^(NH_4_)_2_SO_4_, 0.5 g L^-1 ^MgSO_4_·7H_2_O and concentrations of trace elements and antibiotics similar to starting medium of strategy A. At an OD_600 _of 15 the following nutrient and inducer pulse was added: 170 ml L^-1 ^of feed solution B1 containing 204 g L^-1 ^glycerol, 102 g L^-1 ^tryptone, 6.8 mM IPTG, 6.8 g L^-1 ^L-arabinose and 3 g L^-1 ^MgSO_4_·7H_2_O. After a further cultivation of 4 h a second nutrient and inducer pulse was added: 170 ml L^-1 ^of feed solution B2 containing 204 g L^-1 ^glycerol, 102 g L^-1 ^tryptone and 6.8 g L^-1 ^L-arabinose.

For fed-batch cultivation with two pulses and linear feed (strategy C) the starting medium (V = 1.5 L) was similar to strategy A. At an OD_600 _of 15, a first nutrient pulse without inducers was added: 130 mL L^-1 ^of feed solution C1 containing 248 g L^-1 ^glycerol, 83 g L^-1 ^yeast extract and 165 g L^-1 ^tryptone, 125 mg L^-1 ^ampicillin, 12.5 mg L^-1 ^tetracycline, 100 mg L^-1 ^spectinomycin. At an OD_600 _of 30, a second nutrient and inducer pulse was added: 100 mL L^-1 ^of feed solution C2 containing 240 g L^-1 ^glycerol, 240 g L^-1 ^tryptone, 9.3 g L^-1 ^MgSO_4_·7H_2_O, 120 g L^-1 ^L-arabinose and 12 mM IPTG. At the same time a linear feed was started with a rate of 19 mL L h^-1 ^using feed solution C3 which contained 100 g L^-1 ^h^-1 ^tryptone, 100 g L^-1 ^h^-1 ^L-arabinose, 33 mL L^-1 ^100 × trace elements, 8.2 g L^-1 ^MgSO_4_·7H_2_O, 1 mM IPTG, 67 mg L^-1 ^ampicillin, 6.7 mg L^-1 ^tetracycline and 54 mg L^-1 ^spectinomycin. The total added volume of feed solution C3 was 280-300 mL L^-1^. All starting media, pulse and feed solutions for fed-batch cultivation were sterilized by filtration. Sterile PPG was added directly to the reactor (1 mL L^-1^) to combat foaming. During the induction phase additional PPG was added if required (max. 1 mL L^-1^). Fed-batch cultures were inoculated with uninduced overnight LB shake flask cultures to an OD_600 _of 0.1. The pH was kept at 7.0 (±0.1) by automated addition of 4 M KOH and 20% v/v phosphoric acid. The stirrer speed was set to 1200 rpm and the aeration rate was 0.5-1.0 L_air _L^-1 ^min^-1 ^at the beginning of cultivations. At an OD_600 _above 10 the inflowing air was progressively enriched with pure O_2 _(manual adjustments) in order to keep oxygen saturation between 10 and 100%. Optical density was followed throughout the processes (samples were diluted appropriately with deionized H_2_O) and cell dry weight (CDW) was followed starting from an OD_600 _of around five. Samples for Western blot analysis (total cell protein, see below) were withdrawn from the reactor before induction and in regular intervals after induction directly into ice-cooled beakers.

### Analytical methods

Extracts of periplasmic proteins were prepared by lysozyme treatment [9]. Briefly, cell pellets were resuspended to an OD_600 _of 20 in lysis buffer composed of 30 mM Tris HCl pH 8.0, 1 mM EDTA, 20% w/v sucrose, 1 mg mL^-1 ^lysozyme and Complete protease inhibitor mix (Roche, Basel, Switzerland) and incubated 1 h at 4°C. After centrifugation at 5000 × g and 4°C for 15 min, samples were withdrawn from the supernatant and supplemented with similar volumes of 2 × SDS PAGE sample buffer. For measurements of optical density (600 nm, 1 cm light path) blank values of cuvettes with medium or water were subtracted and samples above an OD_600 _of 0.6 were appropriately diluted with deionized H_2_O. Specific growth rates were calculated by least squares linear regression from linear parts of logarithmic OD_600 _growth curves. Total cell protein (TCP) samples were prepared by resuspending cell pellets obtained from 0.1 to 2 mL culture volume in SDS-PAGE sample buffer to an OD_600 _of 10. Cells were solubilized by heating to ≥ 95°C for 5 min. Glyoconjugates in cell extracts of recombinant *E. coli *were analyzed by SDS-PAGE and subsequent Western blot analysis using Hybond ECL nitrocellulose membranes (GE Healthcare, Waukesha, USA) according to standard procedures. SDS-polyacrylamide gels (10%) used for blotting were always loaded with 10 μL of TCP or 20 μL periplasmic extract per well, i.e., similar volumes of cell extracts originating from similar amounts of biomass were used, enabling a semi-quantitative comparison between samples on the same blot. For detection of AcrA and AcrA-*Shigella *O1 glycoconjugates, rabbit anti-AcrA antibodies were used (described in [8]). EPA and EPA-*Shigella *O1 were detected with a commercial rabbit anti-EPA antiserum (Sigma, Buchs, Switzerland). For specifically detecting glycoproteins, affinity-purified antibodies raised in rabbit against AcrA-*Shigella *O1 glycoconjugates were used (GlycoVaxyn AG, Schlieren, Switzerland). Horseradish peroxidase (HRP)-coupled goat anti-rabbit secondary antibodies (Biorad, Reinach, Switzerland) and SuperSignal™ West Dura HRP substrate (Thermo Fisher Scientific, Rockford, USA) were used for chemiluminescence detection with a ChemiDoc-It imaging system (UVP, Upland, USA). Proteinase K digests of denatured SDS-PAGE protein samples were performed as follows: 50 μL protein samples were supplemented with 1 μL of proteinase K solution (10 mg/mL) and incubated for 1 h at 60°C, followed by incubation for 5 min at 95°C to inactivate the protease.

Cell dry weight was determined by centrifuging 2 mL culture samples (9000 × g, 1 min), resuspending and washing the pellets twice in phosphate-buffered saline and drying the cell pellets for 48 h at 105°C.

## Results

### Glycosylation of AcrA and EPA with *Shigella dysenteriae *type 1 O-specific polysaccharides

**Figure 2:**
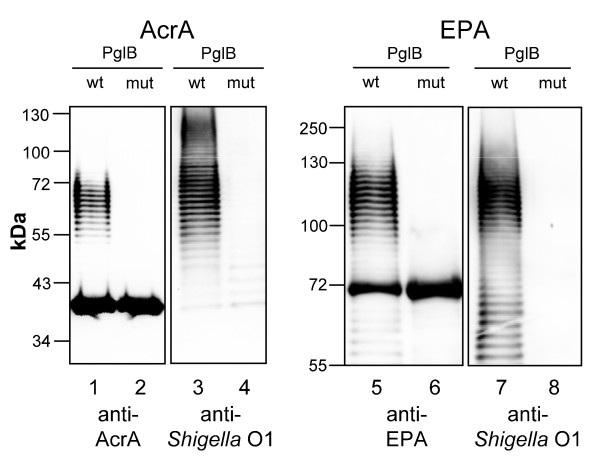
**Glycosylation of AcrA and EPA with *Shigella *O1 polysaccharides**. Extracts of periplasmic proteins from *E. coli *CLM24 expressing carrier protein, *Shigella *polysaccharides (pGVXN64) and either wild-type (wt; pGVXN114) or inactive PglB (mut; pGVXN115) were analysed by Western blot. Lanes 1 and 2: AcrA-expressing strain (pMIK44) analysed with anti-AcrA antibodies; lanes 3 and 4: AcrA-expressing strain analysed with anti-*Shigella *O1 antibodies (same SDS-polyacrylamide gel as lanes 1 and 2); lanes 5 and 6: EPA-expressing strain (pGVXN150) analysed with anti-EPA antibodies; lanes 7 and 8: EPA-expressing strain analysed with anti-*Shigella *O1 antibodies (same SDS-polyacrylamide gel as lanes 5 and 6).

In previous studies it was shown that the glycoprotein AcrA, a periplasmic component of a multidrug efflux pump in *C. jejuni*, was N-glycosylated with *E. coli *O7, *E. coli *9a, *E. coli *O16, *P. aeruginosa *O11 and *C. jejuni *oligo- and polysaccharides in recombinant *E. coli *when functional oligosaccharyltransferase PglB was co-expressed [8,9]. Here we show that AcrA (40 kDa) was glycosylated with *S. dysenteriae *type 1 polysaccharides (*Shigella *O1) in *E. coli *CLM24 [9] containing plasmids pMIK44 (periplasmic AcrA expression), pGVXN114 (PglB expression) and pGVXN64 (gene cluster for *Shigella *O1 synthesis) (Figure 2, lane 1). Glycosylation of AcrA with *Shigella *O1 was abolished when pGVXN114 was replaced by pGVXN115, a plasmid encoding the inactive oligosaccharyltransferase variant PglBmut (Figure 2, lane 2). Antibodies against *Shigella *O1 antigens reacted with glycosylated AcrA while no glycoprotein was detected in periplasmic extract of the PglBmut strain (Figure 2, lanes 3 and 4). The weak bands around 40 kDa visible in lane 4 may be due to contamination with UDP-linked O antigens.

Periplasmically expressed EPA toxoid (69 kDa) containing two engineered N-glycosylation sites on the protein surface (pGVXN150) was also glycosylated with *Shigella *O1 in *E. coli *CLM24 co-expressing PglB and *Shigella *polysaccharide synthesis genes (Figure 2, lane 5). Again, no glycosylated bands were detected in periplasmic extract from cells expressing PglBmut (Figure 2, lane 6). Glycosylated EPA was detected by anti-*Shigella O1 *antibodies while no glycoprotein bands were detected when cells expressed PglBmut (Figure 2, lanes 7 and 8).

Neither AcrA nor EPA present in soluble form in the periplasm was fully glycosylated in recombinant *E. coli *(Figure 2, lanes 1 and 5). Similar to other O-polysaccharide-protein conjugates produced in *E. coli *[9], a ladder of glycoprotein bands appeared in Western blots (Figure 2, lanes 1, 3, 5 and 7). This is indicative of O polysaccharides with different chain lengths generated by the enzymes Wzy and Wzz. Wzy is responsible for the polymerisation of the repeating polysaccharide subunits and Wzz controls the number of polymerization steps in O-antigen synthesis [24]. In the case of AcrA-*Shigella *O1 (AcrA-O1), a second ladder of bands between 100 and 130 kDa could be detected with anti-*Shigella *O1 antibodies, which indicates the formation of diglycosylated AcrA-O1 (Figure 2, lane 3). A second ladder was absent in EPA-*Shigella *O1 (EPA-O1), although two N-glycosylation sites had been introduced. Nevertheless, either site was glycosylated in EPA variants where only one of the two sites was present (to be published elsewhere, manuscript in preparation). This suggests inefficient production of diglycosylated forms of EPA-O1. Ladder-like signals at a molecular weight below 70 kDa detected with anti-EPA and anti-*Shigella *O1 antibodies (Figure 2, lanes 5 and 7) most likely represent proteolytically degraded EPA-O1 as shown by a proteinase K digestion assay (Additional file 1).

### Growth of glycoconjugate producing strains in defined mineral medium

Mineral salts media are preferred for establishing precisely controlled bioprocesses. Therefore, it was tested whether glycoconjugate producing *E. coli *strains can be cultivated in defined mineral medium with glucose as only carbon and energy source. The prototrophic, plasmid-free host strain *E. coli *CLM24 (W3110 Δ*waaL*) grew with a specific growth rate of 0.55 ± 0.02 h^-1 ^at 37°C in glucose mineral medium. However, the specific growth rate of both *E. coli *CLM24 (pMIK44, pGVXN64, pGVXN114) and CLM24 (pGVXN150, pGVXN64, pGVNX114) in glucose mineral medium without inducers was significantly reduced to 0.30 ± 0.01 h^-1 ^and 0.34 ± 0.02 h^-1^, respectively, which is 3- to 4-fold lower than the specific growth rates of these strains in LB complex medium before induction (Table 1). The growth impairment cannot be explained solely by the presence of three antibiotics, as the specific growth rate of CLM24 (pMIK44, pGVXN64, pGVXN114) in antibiotic-free mineral medium was also significantly reduced (0.40 ± 0.01 h^-1^) compared to CLM24. More likely the reduced growth rate was due to a bottleneck in precursor supply for plasmid DNA synthesis [25,26]. To overcome the impaired growth, we added the complex supplements yeast extract and tryptone to medium formulations for subsequent batch, chemostat and fed-batch experiments.

**Table 1:** Effect of induction on specific growth rate (*μ*) of glycoconjugate producing *E. coli *and final optical density in batch and fed-batch culture (ara = L-arabinose)

Product	Type of cultivation	Induction	**OD**_**600**_** at induction**	***μ *before induction (h**^**-1**^**)**	***μ *after induction (h**^**-1**^**)**	**final OD**_**600**_
AcrA-O1	LB batch bioreactor	ara + IPTG	0.5	1.15	0.12	0.9
AcrA-O1	LB batch bioreactor	IPTG	0.5	1.12	0.23	1.3
AcrA-O1	LB batch shake flask	ara + IPTG	0.5	1.08	0.15	1.5-2.0
AcrA-O1	LB batch shake flask	ara	0.5	0.86	0.56	2.2
AcrA-O1	Fed-batch strategy A	ara + IPTG	47	0.24	0.08	58
AcrA-O1	Fed-batch strategy B	ara + IPTG	14	0.55	0.16	59
AcrA-O1	Fed-batch strategy C	ara + IPTG	31	0.30	0.13	42

EPA-O1	LB batch shake flask	ara + IPTG	0.5	0.92	0.18	1.9
EPA-O1	Fed-batch strategy C	ara + IPTG	35	0.60	0.12	78

### Glycoconjugate formation in LB batch culture and effect of induction on growth

**Figure 3:**
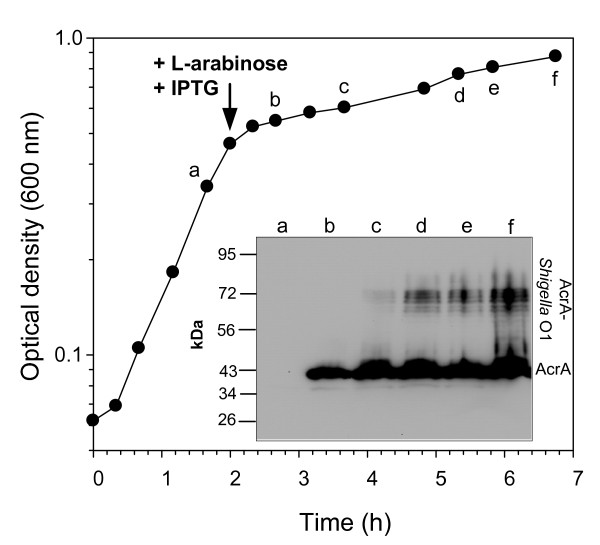
**Growth and glycoconjugate formation in batch culture**. AcrA-O1 producing *E. coli *CLM24 (pMIK44, pGVXN64, pGVXN114) were cultivated in LB medium in a 2 L-bioreactor. The arrow indicates the time point of induction with 2 g L^-1 ^L-arabinose and 1 mM IPTG. Normalized total cell protein samples were taken at the indicated time points (a to f) and analysed by Western blot using anti-ArcA antibodies.

Previously, glycoconjugates in recombinant *E. coli *were produced in shake flask cultures. For a more efficient production cultivation in bioreactors was required. As a first step, glycoconjugates were produced in a 2 L scale batch culture in a fully aerated bioreactor using LB medium. Formation of AcrA-O1 was observed 5 h after simultaneous induction of gene expression for the carrier protein and PglB (Figure 3). Neither AcrA nor AcrA-O1 were present in detectable amounts before induction (Figure 3, lane a). While AcrA appeared in less than 1 h after induction, glycosylated AcrA was detected not before 4 hours of induction (Figure 3, lanes b to f).

Induction of glycoconjugate synthesis caused a strong growth inhibition of the production strain (Figure 3). Immediately after induction, the specific growth rate dropped by a factor of 10 (Table 1). A similar effect was observed in LB shake flasks (Table 1), while growth in uninduced shake flasks gradually slowed down between an OD_600 _of 0.5 an 1.5 (data not shown), which is typical for *E. coli *growing in LB medium [27]. In order to analyse which of the two recombinant proteins was responsible for growth inhibition, LB shake flask cultures were induced with either 2 g L^-1 ^L-arabinose (*acrA *expression) or 1 mM IPTG (*pglB *expression) at an OD_600 _of 0.5. While the specific growth rate after induction with L-arabinose was similar to that of uninduced cultures, induction with only IPTG lead to a similarly strong growth inhibition as when both inducers were added (Table 1). Specific growth rates of the EPA-O1 producing strain before and after induction in LB batch culture were similar to the AcrA-O1 producing strain (Table 1). The initial specific growth rate of plasmid-free *E. coli *CLM24 in LB batch culture (1.3 ± 0.04 h^-1^, n = 3) was only slightly higher than that of uninduced strains bearing plasmids for glycoconjugate production (Table 1).

We found that glycoconjugate yield in LB shake flask cultures was higher when the induction period was extended to 20 h and when non-baffled flasks and a low surface to volume ratio were used (70 mL in 100 mL flasks). Therefore, such cultures were used as benchmark for glycoconjugate formation in fed-batch cultures.

### Effect of reduced IPTG concentrations and low cultivation temperature

Given the growth inhibitory effect of IPTG-induced expression of *pglB*, it was tested whether the use of reduced IPTG concentrations and/or reduced cultivation temperature enhances the yield of glycoconjugates in *E. coli*. Low IPTG concentrations and reduced growth temperature have been shown to increase the yield of correctly folded membrane proteins expressed from P_*lac*_/P_*tac *_controlled plasmids [28,29]. However, neither of the two changes increased the yields of glycoprotein (Additional file 2). A stepwise reduction of IPTG from 1 mM to 5 μM marginally affected the level of AcrA-O1 and EPA-O1. The use of a lower cultivation temperature before induction (30°C) and switch to room temperature (23°C) at induction was also not beneficial. The level of AcrA-O1 was significantly reduced, while the amount of synthesized EPA-O1 seemed not to be affected (Additional file 2). Interestingly, glycoprotein bands were shifted on average to higher molecular weights at reduced cultivation temperature (Additional file 2). Due to the absence of strong positive effects of reduced inducer concentrations or lower temperatures, an IPTG concentration of 1 mM and a cultivation temperature of 37°C were used for subsequent experiments.

### Effect of carbon- and energy sources on the efficiency of glycosylation

Chemostat experiments were performed in order to evaluate the suitability of additional carbon- and energy sources for glycoconjugate production with increased biomass yield. Chemostat cultivation allows to change a single factor (medium composition) while keeping all other culture parameters the same. Dilution rate equals specific growth rate and steady state biomass concentration is determined by the concentration of the limiting nutrient(s) in the feed [30]. To avoid accumulation of excess nutrients and washing out of cells during induction, a dilution rate of 0.1 h^-1 ^was chosen, which is lower than the specific growth rate observed during the induction phase in LB batch cultures (Table 1). A sequential induction strategy with 4 h L-arabinose wash-in (0.2 g L^-1 ^h^-1^) followed by an IPTG pulse (1 mM) was used in order to reduce stress caused by recombinant protein production.

**Figure 4:**
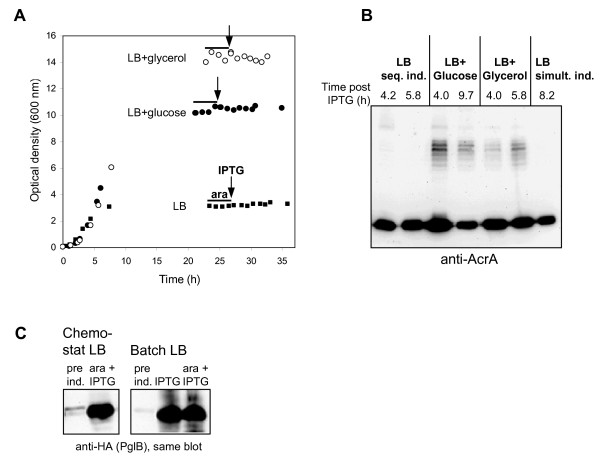
**Chemostat cultivation (*D *= 0.1 h^-1^) of AcrA-O1 producing *E. coli *using different growth substrates**. (A) Time course of optical density after inoculation, bars indicate the period of L-arabinose (ara) co-feed and arrows indicate the time point of the addition of 1 mM IPTG. (B) Glycoconjugate formation in chemostat cultures analysed with anti-AcrA antibodies on Western blot (normalized samples). (C) Expression of *pglB *in LB chemostat culture compared to batch culture (anti-HA Western blot, normalized samples).

Chemostats were induced after two volume changes in order to keep the cultivation time before induction as short as possible as a precaution against genetic changes (e.g., plasmid loss). Although this is somewhat lower than the usually recommended 3-5 volume changes, constant OD_600 _values during the induction period indicated that the cultures had reached a steady state (Figure 4A). In chemostat culture with LB medium (15 g L^-1 ^of organic nutrients) a steady state OD_600 _of 3.2 was reached (Figure 4A). Steady state optical densities were increased by a factor of three to four in glucose-LB and glycerol-LB chemostats (Figure 4A), in spite of lower total amounts of organic nutrients in the feed medium (13 g L^-1^). AcrA was produced after the start of an L-arabinose co-feed in chemostat cultures with all three media (Figure 4B, strong, lower bands). This indicates that the cells were growing under carbon- and energy-limited conditions because the P_*BAD *_promoter, which controls *acrA *expression from plasmid pMIK44, is highly sensitive to catabolite repression [31].

In spite of strong expression of *acrA*, no formation of AcrA-O1 glycoconjugates could be detected within 6 h of *pglB *induction in LB medium, both with sequential and simultaneous addition of L-arabinose and IPTG (Figure 4B). The inefficient glycosylation was not due to reduced or absent production of PglB. Strong bands with the size of PglB were detected with anti-HA antibodies in the LB chemostat after induction (Figure 4C). The intensity of these bands was comparable to that obtained with fast growing LB batch cultures that had been induced either with IPTG alone or with IPTG and arabinose at an OD_600 _of 0.5 (Figure 4C). Significant glycoconjugate synthesis was detectable when either glucose or glycerol were added to LB medium as main carbon- and energy source in chemostat cultures (Figure 4B). In summary, with the use of either glucose or glycerol as main carbon- and energy source in semi-defined media it was possible to reach 5-10 fold higher biomass concentrations and at the same time keeping a reasonable efficiency of glycosylation

### Production of glycoconjugates in fed-batch culture

Based on results from batch and chemostat experiments, a semi-defined medium with glycerol, yeast extract and tryptone was chosen for fed-batch culture. We used glycerol instead of glucose as main carbon- and energy source to avoid interferences with the glucose sensitive P_*BAD *_promoter. In contrast to glucose, levels of cAMP are high and acetate accumulation is low even if excess amounts of glycerol are present in *E. coli *cultures [32,33].

**Figure 5:**
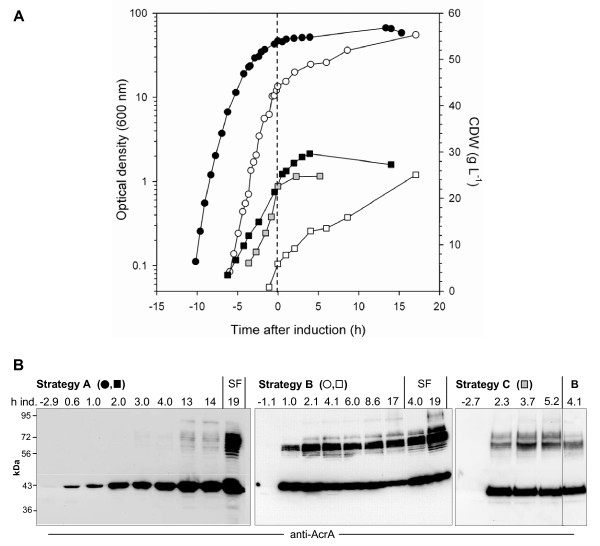
**Fed-batch cultivation of AcrA-O1 producing *E. coli *with semi-defined glycerol medium using three different feed and induction strategies**. Strategy A - filled symbols: Linear feed of glycerol and tryptone from time -3 to 0 h, pulse of IPTG (1 mM) and L-arabinose (2 g L^-1^) at time 0 h, linear feed of glycerol, tryptone, L-rabinose (2.75 g L^-1 ^h^-1^) and IPTG (80 μM h^-1^) from time 0 to 15 h. Strategy B - open symbols: Pulse of glycerol, tryptone, L-arabinose (4 g L^-1^) and IPTG (1 mM) at time 0 h, pulse of glycerol, tryptone and L-arabinose (4 g L^-1^) at time 4 h. Strategy C - shaded symbols: Pulse of glycerol, yeast extract and tryptone at time -2.6 h; pulse of glycerol, tryptone, L-arabinose (10 g L^-1^) and IPTG (1 mM) at time 0 h; linear feed of tryptone, L-arabinose (1.6 g L^-1 ^h^-1^) and IPTG (10 μM h^-1^) from time 0 to 15 h. (A) Logarithmic growth curve (circles) and time course of biomass concentrations (squares), time 0 h (broken line): induction with L-arabinose and IPTG. (B) Time course of AcrA and AcrA-O1 formation. Normalized total cell protein samples were analysed by Western blot with anti-AcrA antibodies, numbers indicate time after induction, SF: samples from LB shake flask cultures. Lane B: same sample of strategy B as in middle blot, analysed on a blot with samples of strategy C (shorter development time, non-relevant lanes removed). *E. coli *CLM24 (pMIK44, pGVXN64, pGVXN114).

Two fed-batch strategies were evaluated first: linear nutrient feed (strategy A) and two consecutive nutrient and inducer pulses (strategy B). With both strategies, 30-fold higher final optical densities (600 nm) compared to LB shake flask cultures were reached for the AcrA-O1 producing strain (Table 1, Figure 5A). However, strategy A with induction at an OD_600 _of 47 failed to yield sufficient amounts of glycosylated protein (Figure 5B). In contrast, Strategy B with induction at an OD_600 _of 14 yielded AcrA-O1 in comparable amounts as in LB shake flask (Figure 5B). A possible factor which influenced glycosylation was the specific growth rate at induction, which was 2-fold lower with strategy A compared to strategy B (Table 1, see also logarithmic growth curves in Figure 5A). Strategy C with induction at an intermediate cell density (OD_600 _= 30) and addition of two nutrient pulses followed by a linear feed of L-arabinose and tryptone lead to the highest levels of glycosylation in fed-batch culture (Figure 5B). In contrast to strategy A, antibiotics were included in the linear feed of strategy C which might have improved plasmid retention, and thus glycoconjugate formation.

**Figure 6:**
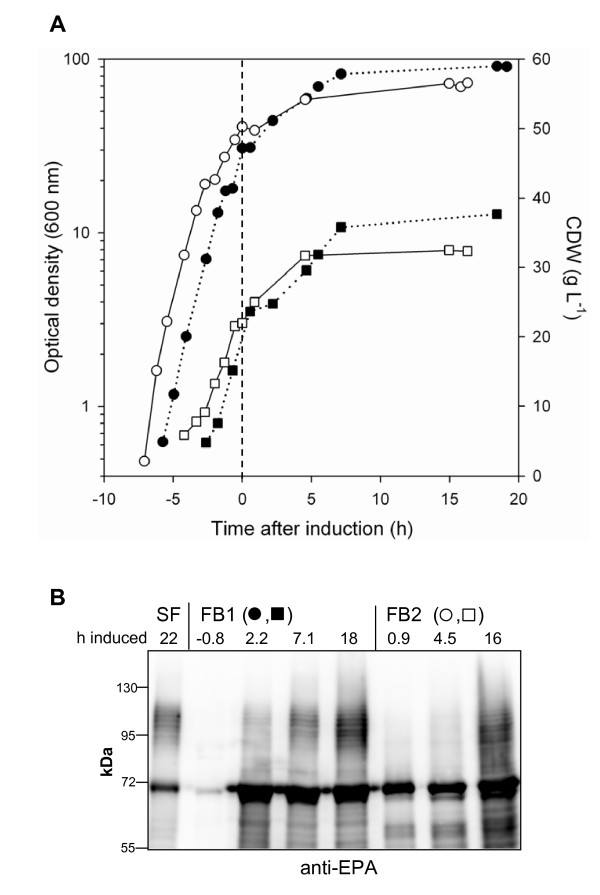
**Production of EPA-O1 in fed-batch culture**. Cultivation and induction were performed according to strategy C as described in Materials and methods and legend to Figure 5. Strain: *E. coli *CLM24 (pGVXN150, pGVXN64, pGVXN114). (A) Logarithmic growth curve (circles) and time course of biomass concentrations (squares). Filled symbols, dotted line: fed-batch run 1 (FB1); open symbols, solid line: fed-batch run 2 (FB2). Time 0 h (broken, vertical line): induction with L-arabinose and IPTG. (B) Time course of EPA and EPA-O1 formation in fed-batch culture compared to LB shake flask culture (SF); anti-EPA Western blots, normalized samples, numbers indicate time after induction.

Fed-batch strategy C was then tested for production of the second glycoconjugate EPA-O1. Two independent fed-batch runs yielded similar levels of glycoprotein as found in LB shake flask cultures after a total cultivation time of 25 h (Figures 6A and 6B). Final OD_600 _was increased from 2 in shake flask to 80 in fed-batch culture (Table 1), thus volumetric productivity was increased by a factor of 40. Glycoprotein EPA-O1 formation in fed-batch culture was seen with a more pronounced retardation compared to cultures producing AcrA-O1 (Figures 5B and 6B). This difference was not due to reduced or delayed expression of the carrier protein (Bands 40 kDa in Figure 5B and bands 70 kDa in Figure 6B). Similarly to batch cultures, induction of carrier protein and PglB synthesis in fed-batch cultures lead to an immediate reduction of the specific growth rate (Figures 5A and 6A, Table 1).

The bands below the size of EPA which were detected in total cell protein samples from induced cultures most likely represent truncated, glycosylated and/or unglycosylated EPA. The bands completely disappeared after treatment with proteinase K (Additional file 1).

AcrA-O1 was purified from LB shake flask cultures with a yield of 0.6 mg L^-1 ^by using osmotic shock extraction followed by Ni-NTA affinity and fluoroapatite chromatography (GlycoVaxyn AG, unpublished results). Based on comparable glycoconjugate yields per cell, but a 30- to 40-fold increase of final cell density with the described fed-batch procedure, 18 to 24 mg L^-1 ^of purified bioconjugate against *Shigella dysenteriae *type 1 can be produced with the current *E. coli in vivo *system (productivity based on total process time: 0.75 to 1.0 mg L^-1 ^h^-1^).

## Discussion

The exploitation of protein glycosylation in recombinant *E. coli *promises to significantly simplify the production of conjugate vaccines (Figure 1). The scope of this technology benefits from the relaxed substrate specificity of the key enzyme PglB, as not only *Campylobacter *oligosaccharides (the natural substrates) are N-linked to the carrier protein AcrA but also several O antigens from other Gram-negative bacteria [9]. Our results show that *S. dysenteriae *type 1 oligosaccharides with the repeating unit →3)-α-L-Rha*p*-(1→3)-α-L-Rha*p*-(1→2)-α-D-Gal*p*-(1→3)-α-D-Glc*p*NAc-(1→ [34] are another suitable substrate for PglB, resulting in periplasmic biosynthesis of AcrA-*Shigella *O1 glycoconjugates. Similarly to the efficiently transferred *E. coli *O7 polysaccharides [9], *Shigella *O1 repeating units contain an N-acetylglucosamine sugar at the reducing end which is α(1→3)-linked to galactose. Until now, all oligo- and polysaccharides which were successfully linked to carrier proteins had an N-acetyl-sugar moiety at the reducing end, presumably due to a participation of the acetyl group in the catalytic mechanism [10]. For the production of conjugate vaccines in *E. coli *it is desirable to have alternatives to AcrA as carrier protein. Here we show that engineered *P. aeruginosa *exotoxoid A (EPA) is also efficiently glycosylated *in vivo *with *S. dysenteriae *type 1 polysaccharides when expressed in the periplasm of recombinant, glycosylation-competent *E. coli*. EPA had been used successfully by others as immunogenic carrier protein in a chemically coupled conjugate vaccine against *Shigella flexneri *type 2a [2].

Overexpression of recombinant proteins in *E. coli *is often associated with negative effects on host physiology, reflected in a reduced specific growth rate, reduced respiratory capacity, increased levels of alarmones and upregulated stress defence genes [35-38]. In our study we observed a considerable metabolic burden already in uninduced cells. This could be alleviated by including complex supplements in the medium. In spite of optimal, surplus nutrient supply in batch and fed-batch culture, growth was strongly inhibited after induction of oligosaccharyltransferase PglB. Growth inhibition presumably was due to stress caused by aggregated, misfolded proteins [38]. PglB is an integral membrane protein [9] and such proteins are prone to inclusion body formation and often severely affect cell physiology when overexpressed in recombinant *E. coli *[39]. Reduced inducer concentrations and/or lower cultivation temperature in many cases improve the expression of correctly folded "problematic" recombinant proteins [28,29,40]. However, in our case no significant improvements could be obtained with these strategies. The vector pEXT21 used for PglB expression is a low copy number plasmid. When fully induced with 1 mM IPTG, expression levels are as low as for a high copy number, pBR322-derived plasmid induced with only 20 μM IPTG [21]. In our experiments the use of 50, 20 and 5 μM IPTG should have reduced the expression level of PglB by 25, 60 and 90%, respectively [21]. This further reduction did not seem to have an additional positive effect compared to the reduced expression level already achieved by using the low copy number vector.

The results obtained in this study demonstrate the suitability of a vector combination with independent, tight control of PglB and carrier protein expression for efficient glycoconjugate production with *E. coli*. P_*BAD *_is well-known for its tight repression [31] while P_*tac *_is prone to leaky expression if LacI repressor levels are too low [41]. In our case, with the gene for LacI present on the pEXT21-derived plasmid, no expression was detectable in the absence of inducers for both promoters (Figures 3 and 4C).

Several novel aspects of the *E. coli in vivo *system for glycoprotein production became apparent. First, the delayed appearance of glycoconjugates on immunoblots compared to the carrier proteins indicates that N-glycosylation was the rate-limiting step, assuming that polysaccharide precursors were not limiting. Second, slight differences in cultivation conditions such as presence of an additional carbon source or specific growth rate at induction had an unexpectedly strong effect on the efficiency of glycosylation, but carrier protein synthesis remained unaffected. Pre-induction growth rates have been shown to influence the yields of recombinant proteins in other studies [42,43], but mostly only one protein had to be expressed. A systematic analysis of how cell physiology influences the efficiency of glycosylation would be of high practical relevance, but was without the scope of this study.

In this work it was demonstrated for the first time that glycosylated proteins can be produced in recombinant *E. coli *at a larger scale in fed-batch culture. We used a semi-defined glycerol medium and a simple pulse feed strategy. Such a design had been used successfully by others in *E. coli *processes, e.g., for the production of bovine growth hormone [44]. It is clear that the time-space-yields for glycoconjugates produced in *E. coli *are still low compared to unmodified, cytoplasmic recombinant proteins for which productivities up to 0.2 g L^-1 ^h^-1 ^and final yields of several grams per litre can be reached [43-46]. Yet, it has to be taken into account that the amounts of glycoconjugate needed per vaccination are rather low (25-100 μg, [2,47]). Most likely, the yields of glycoconjugates in *E. coli *can be further increased using the following strategies: First, additional process modifications might lead to more complete glycosylation of carrier proteins. Second, alternative *E. coli *host strains could be tested, e.g., "Walker" strains which are known for improved membrane protein expression [48]. Third, if metabolic bottlenecks for glycosylation such as precursor supply could be identified, it would be possible to improve host strains accordingly by molecular engineering. Fourth, functional expression and/or catalytic efficiency of PglB could be improved by protein engineering. Finally, the sequence context of glycosylation sites in carrier proteins could be further optimized e.g., by creating surface-exposed loops with higher flexibility [12].

## Conclusions

This study shows how conjugate vaccines can be produced in recombinant *E. coli *in a simple and cost-efficient way superior to state-of-the-art chemical coupling technologies. The potential of the *in vivo *process is underlined by the successful coupling of O antigen polysaccharides of the important intestinal pathogen *Shigella dysenteriae *to two different carrier proteins. By using appropriate growth and induction conditions in fed-batch culture it was possible to increase the productivity for *in vivo *synthesis of conjugate vaccines by a factor of 40 compared to shake flask culture, facilitating an economically viable production.

## Competing interests

The authors declare that they have no competing interests.

## Authors' contributions

JI designed and analyzed the cultivation experiments and was involved in performing them and he drafted the manuscript. MK and MW designed and constructed the expression vectors and contributed to the process design. SD performed most of the bioreactor experiments and was involved in data analysis. CT carried out most of the Western blot analyses. LTM participated in setting up and designing the study and contributed to data interpretation and drawing conclusions. MK, MW and LTM helped to draft the manuscript. All authors read and approved the final manuscript.

## Supplementary Material

Additional file 1 **Degradation of EPA and EPA-O1 by proteinase K**. SDS-PAGE samples were taken from induced, overnight LB batch cultures of EPA-O1 producing *E. coli *(PglB wt) and a PglBmut control strain. Aliquots were subjected to treatment with proteinase K for 1 h at 60°C (Proteinase K +). (A) Periplasm extract and total cell protein samples analysed with anti-EPA antibodies on Western blot. (B) Periplasm extract and total cell protein samples analysed with anti-*Shigella *O1 antibodies. Click here for file

Additional file 2 **Effect of IPTG concentration and cultivation temperature on AcrA-O1 and EPA-O1 formation**. Normalized total cell protein samples were taken from L-arabinose and IPTG induced LB shake flask cultures after overnight incubation and analysed by Western blot using anti-ArcA and anti-EPA antibodies. (A) AcrA-O1 producing *E. coli *CLM24 (pMIK44, pGVXN64, pGVXN114). (B) EPA-O1 producing *E. coli *CLM24 (pGVXN150, pGVXN64, pGVXN114). Click here for file
