# Supplementary figures and images for: Production of glycoprotein vaccines in Escherichia coli
Source: Microb Cell Fact. 2010 Aug 11;9:61. doi: 10.1186/1475-2859-9-61 (PMC2927510; doi:10.1186/1475-2859-9-61)

A

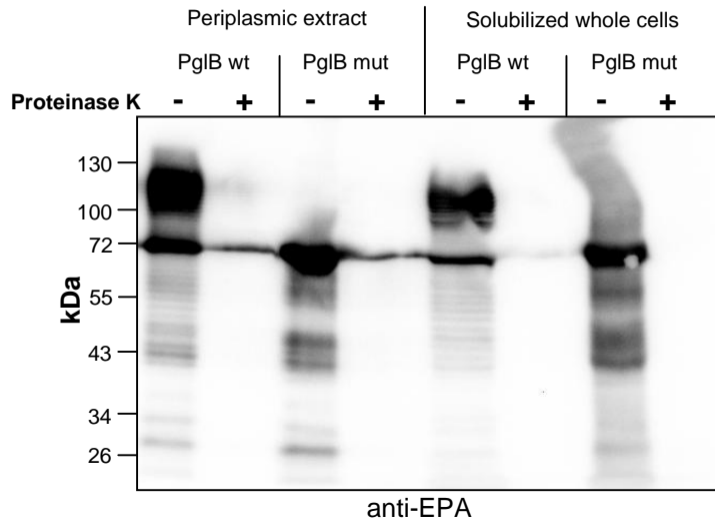

B

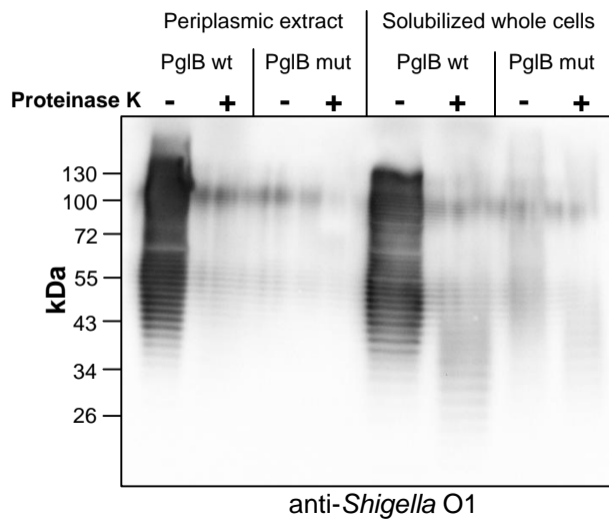

Supplement: Additional file 1 — Degradation of EPA and EPA-O1 by proteinase K. SDS-PAGE samples were taken from induced, overnight LB batch cultures of EPA-O1 producing E. coli (PglB wt) and a PglBmut control strain. Aliquots were subjected to treatment with proteinase K for 1 h at 60°C (Proteinase K +). (A) Periplasm extract and total cell protein samples analysed with anti-EPA antibodies on Western blot. (B) Periplasm extract and total cell protein samples analysed with anti-Shigella O1 antibodies. [file 1475-2859-9-61-S1.PDF]

**A**

37°C

30&gt;23°C

IPTG ( $\mu$ M) 1000 50 20 5 | 1000 50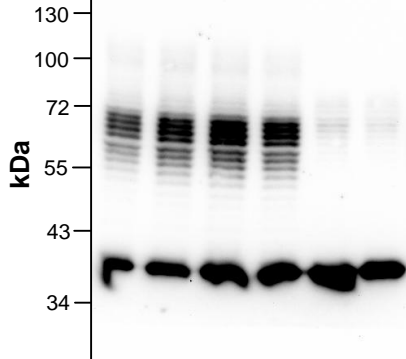

anti-AcrA

**B**

37°C

30&gt;23°C

IPTG ( $\mu$ M) 1000 50 20 5 | 1000 50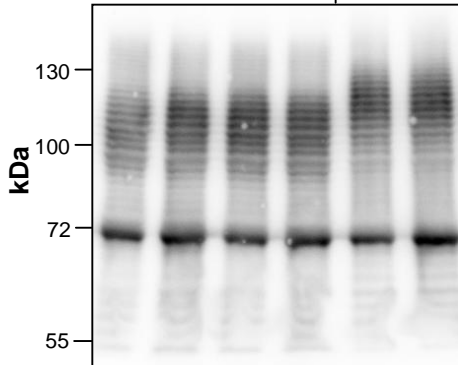

anti-EPA

Supplement: Additional file 2 — Effect of IPTG concentration and cultivation temperature on AcrA-O1 and EPA-O1 formation. Normalized total cell protein samples were taken from L-arabinose and IPTG induced LB shake flask cultures after overnight incubation and analysed by Western blot using anti-ArcA and anti-EPA antibodies. (A) AcrA-O1 producing E. coli CLM24 (pMIK44, pGVXN64, pGVXN114). (B) EPA-O1 producing E. coli CLM24 (pGVXN150, pGVXN64, pGVXN114). [file 1475-2859-9-61-S2.PDF]
